# Collection of analog series-based scaffolds from public compound sources

**DOI:** 10.4155/fsoa-2017-0135

**Published:** 2018-02-08

**Authors:** Dilyana Dimova, Jürgen Bajorath

**Affiliations:** 1 Department of Life Science Informatics, B-IT, LIMES Program Unit Chemical Biology & Medicinal Chemistry, Rheinische Friedrich-Wilhelms-Universität, Dahlmannstr. 2, D-53113 Bonn, Germany

**Keywords:** analog series, ASB scaffolds, bioactive compounds, computational analysis, open access data, scaffold database

## Abstract

**Aim:**

Providing a large and freely available *in silico* collection of analog series-based (ASB) scaffolds for computational design and medicinal chemistry applications.

**Methodology:**

Using a recently introduced computational method, ASB scaffolds with single and multiple substitution sites were systematically isolated from publicly available active compounds.

**Data:**

A total of 23,791 unique ASB scaffolds are made available in an organized and machine-readable form as an open access deposition. For each ASB scaffold, the number of analogs it represents is also provided.

**Next steps:**

The freely available collection of ASB scaffolds will be further analyzed to explore different types of scaffold relationships. The ASB scaffold collection will periodically be updated.

## Lay abstract

The scaffold concept is popular in medicinal chemistry, drug design and chemical informatics. Scaffolds represent core structures of small molecules. They can be defined and computationally generated in different ways. Scaffolds extracted from individual compounds are distinguished from scaffolds representing series of closely related compounds, so-called analogs. Herein, a large collection of analog series-based scaffolds is reported. These scaffolds are made freely available to aid in medicinal chemistry and drug design applications.

## Graphical abstract

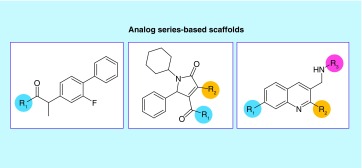

Analog series-based scaffolds were systematically isolated from public compound sources to generate a comprehensive and freely available scaffold collection. The figure shows exemplary analog series-based scaffolds. Substitution sites are color-coded.

**Key terms**

**Analog series (AS):** Series of closely related compounds sharing the same core structure and having different substituents at one or more sites.

**Analog series-based (ASB) scaffold:** Scaffolds derived from analog series (instead of individual compounds) taking chemical reaction information into account.

**Matched molecular pair (MMP):** A pair of compounds that are distinguished by a chemical modification at a single site.

**Scaffold:** A term describing the core structure of a compound or series.

**Substituent:** Chemical moiety (functional group, R-group) attached to a scaffold. Analogs belonging to a series are distinguished by different substituents at given site(s).

In medicinal chemistry and chemical informatics, the term ‘scaffold’ conventionally refers to core structures of compounds [1,2]. According to the first generally applicable definition, a scaffold was obtained from a compound by removing all substituents from rings and substructures connecting rings [3]. By definition, a so-defined scaffold consisted of one or more remaining ring systems (i.e., a molecule containing no ring did not yield a scaffold). This compound-based scaffold definition provided a basis for the computational analysis of core structures. Scaffold analysis has mostly focused on active compounds, for example, to organize them according to different core structures, analyze structure-activity relationships (SARs), provide templates for molecular design, or search for compounds with different structures but similar activity [1,2]. Furthermore, scaffolds are often annotated with target information of compounds they represent. This provides a meta-level assignment of biological activities of compounds to core structures that can then be used, for example, as templates for target-directed molecular design [1]. We have recently developed an alternative to compound-based scaffold definitions. Following our approach, scaffolds are derived from analog series (ASs), rather than individual compounds, leading to the introduction of analog series-based (ASB) scaffolds [4,5]. ASB scaffolds were designed to incorporate synthetic information, contain single or multiple substitution sites, and capture all structural features conserved in an AS. These design elements were thought to further increase the utility of computationally generated scaffolds for medicinal chemistry applications. Importantly, a given AS yields one and only one ASB scaffold, which is meaningful chemically, whereas it might yield multiple compound-based scaffolds. Furthermore, ASB scaffolds generally capture more series-specific SAR and target information than compound-based scaffolds. By design, ASB scaffolds are distinct from conventional compound-based scaffolds [4]. Studies reporting the extraction, organization, analysis and application of compound based-scaffolds have been reviewed extensively [1,2].

Herein, we report the systematic extraction of ASB scaffolds from public repositories of bioactive compounds and the generation of a comprehensive open access collection of ASB scaffolds.

## Methodology

### Generation of ASB scaffolds

In the following, a summary of the ASB scaffold approach is provided. For further methodological details, the interested reader is referred to the original open access publications introducing first- [4] and second-generation ASB scaffolds [5]. The derivation of ASB scaffolds is a two-stage process. In stage one, ASs are extracted from compound data sets using a computational method specifically developed for this purpose [6]. This method adapted the matched molecular pair (MMP) [7] formalism to exhaustively account for pairwise analog relationships as a prerequisite for identifying unique ASs. An MMP is defined as a pair of compounds that are only distinguished by a chemical modification at a single site [7]. Following our approach, MMPs were computationally generated by molecular fragmentation on the basis retrosynthetic rules [8] producing RECAP-MMPs (RMMPs) [8]. An RMMP consists of a conserved core structure and a pair of exchanged substituents. Chemical modifications in RMMPs were size-restricted to limit substitutions to those found in ASs from medicinal chemistry [9].

In stage two, all possible RMMP cores of an AS were searched for a core shared by all analogs comprising the series. Thus, this RMMP core was required to capture all pairwise RMMP relationships within the AS. If such a core was identified, it was defined as the ASB scaffold representing the series [4]. By design, these first generation ASB scaffolds were only extracted from ASs having a single substitution site. This limited the ASs coverage of ASB scaffolds to about 70% of series extracted from the ChEMBL database (releases 21 and 22) [10]. Therefore, the ASB method was further extended to generate ASB scaffolds from ASs with multiple substitution sites [5]. Methodological modifications were introduced to identify nonredundant RMMP cores for ASs and map multiple substitution sites represented by different nonredundant RMMP cores to a single ASB scaffold [5]. This further increased the ASs coverage of ASB scaffolds to more than 90% for ChEMBL compounds (release 22) [5].

**Figure 1.**
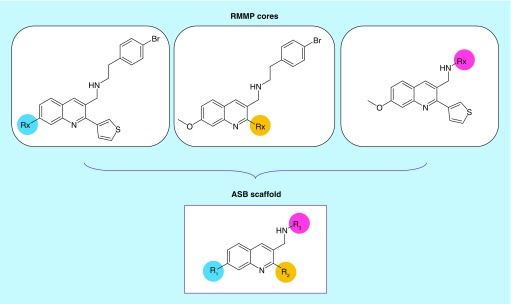
**Exemplary analog series-based scaffold.** An ASB scaffold is shown that was extracted from an AS containing three RMMP cores. Substitution sites are denoted R_x_ in cores and R_n_ in the resulting ASB scaffold and color-coded. AS: Analog series; ASB: Analog series-based; RMMP: RECAP-matched molecular pair.

**Figure 2.**
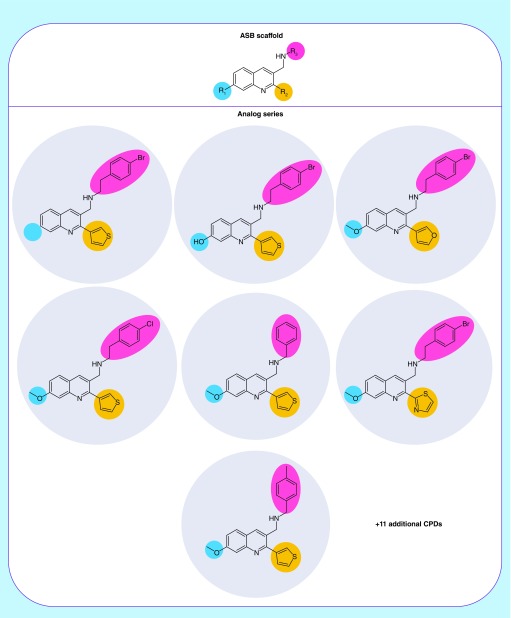
**Exemplary analog series-based scaffold.** Seven exemplary analogs are shown from the analog series represented by the ASB scaffold. In these analogs, R-groups at the three substitution sites are highlighted. ASB: Analog series-based; CPD: Compound.

Figure 1 shows an exemplary ASB scaffold generated from multiple RMMP cores of an AS representing different substitution sites. Figure 2 shows analogs represented by this ASB scaffold.

### Searching for ASB scaffolds

ASB scaffolds with single or multiple substitution sites were systematically extracted from ChEMBL [10], the major public repository of compounds from the medicinal chemistry literature and patents, and a recently introduced database focused on drugs and probes for chemical biology, the Probes & Drugs Portal (PDP) [11]. From ChEMBL (release 23), compounds with available high-confidence activity data were selected using an established data curation protocol [4]. From qualifying compounds, ASs were systematically extracted and subjected to ASB scaffold analysis. Structures from PDP were assembled and standardized using an in-house protocol, ASs were extracted, and ASB scaffolds generated.

## Data

### ASB scaffold collection

The results of our systematic search calculations are summarized in Table 1. From ChEMBL (release 23) and PDP, 23,926 and 2022 ASs were extracted, which yielded 22,094 and 1939 ASB scaffolds, respectively. Thus, from 92.3 and 95.9% of the ASs from these different compound sources, ASB scaffolds were successfully isolated, hence providing extensive ASs coverage. Each ASB scaffold from ChEMBL was annotated with the targets against which the analogs it represented were active and the number of unique targets was determined. This resulted in 12,536 ASB scaffolds with single (56.7%) and 9558 scaffolds with multiple target annotations. In total, the 22,094 ASB scaffolds were annotated with 1473 human targets. Thus, target coverage of systematically identified ASB scaffolds was also extensive. Compounds taken from PDP were designated as drugs, probes or alerts following the PDP-internal classification scheme. If no annotation was available for a given compound it was classified as ‘other’. Multiple annotations for a compound were possible (e.g., alert and drug). In total, there were 1098 ASB scaffolds representing at least one drug and 512 scaffolds exclusively representing drugs. In addition, 122 ASB scaffolds were annotated with alerts representing a total of 406 compounds.

**Table 1.** **Analog series-based scaffold statistics.**

**Database**	**PDP**	**ChEMBL**
ASB scaffolds (n)	1939	22,094

ASs coverage (%)	95.9	92.3

Unique ASB scaffolds (n)	23,791	

ASBs with MT annotations (%)	40.2	

Targets covered (n)	1473	

Shared ASB scaffolds (n)	242	

ASBs with MT annotations (%)	78.9	

Targets covered (n)	531	

The table reports statistics for ASB scaffolds generated from ASs of compounds from PDP and ChEMBL (version 23). The total number of ASB scaffolds and the percentage of ASs yielding ASB scaffolds (% ASs coverage) are provided. In addition, the number of unique and shared ASB scaffolds is given. Furthermore, the percentage of combined ASB scaffolds with MT annotations and total number of corresponding targets is given.

AS: Analog series; ASB: Analog series-based; MT: Multitarget; PDP: Probes & Drugs Portal.

Furthermore, 242 ASB scaffolds were common to ChEMBL and PDP. Hence, a total of 23,791 unique ASB scaffolds were obtained, 84.4% of which contained single and 15.6% multiple substitution sites.

### Open access deposition

The entire collection of 23,791 unique ASB scaffolds is made available in a single file. Each ASB scaffold is provided in canonical SMILES representation [12], substitution sites are designated, the database origin is specified, and the number of analogs each scaffold represents is reported. In addition, for each ASB scaffold from ChEMBL, unique target annotations of corresponding analogs are provided using UniProt target identifiers [13]. For ASB scaffolds from PDP, collected annotations are provided. Scaffolds are rank-ordered according to number of analogs they represent. The ASB-scaffold database is made freely available as a deposition on the ZENODO open access platform [14].

## Limitations & next steps

ASB scaffolds are currently not obtained from a small proportion of ASs (less than 10%) containing multiple sub-series with different substitution sites, which cannot be unambiguously mapped to a single ASB scaffold. Such highly complex ASs are further investigated to generate defined ASB scaffolds. In addition, we continue to analyze our ASB scaffold collection to explore structure and activity relationships between scaffolds. First attempts have also been made to use ASB scaffolds for systematically deriving target hypotheses for phenotypic screens [15]. Furthermore, it is planned to apply the ASB scaffold concept for automated compound design. The open access deposition of ASB scaffolds makes it possible to investigate them for other applications. It is hoped that this will further increase the awareness and utility of ASB scaffolds. The systematic search for ASB scaffolds will periodically be repeated and the deposition updated when significant numbers of new scaffolds become available.

**Executive summary**

- The scaffold concept for computational and medicinal chemistry is discussed.
- Analog series-based (ASB) scaffolds are introduced.

**Methodology**

- A summary of the computational approach to generate ASB scaffolds is provided.
- The systematic search for ASB scaffolds in two compound repositories is detailed.

**Data**

- Results of our large-scale analysis of ASB scaffolds are reported.
- An open access deposition of the ASB scaffold collection is generated and described.

**Limitations & next steps**

- Further applications of ASB scaffolds are discussed.
- The systematic search for ASB scaffolds will periodically be repeated and the open access deposition of ASB scaffolds updated.
